# Prognostic value of thyroid hormones in acute ischemic stroke – a meta analysis

**DOI:** 10.1038/s41598-017-16564-2

**Published:** 2017-11-24

**Authors:** Xingjun Jiang, Hongyi Xing, Jing Wu, Ruofei Du, Houfu Liu, Jixiang Chen, Ji Wang, Chen Wang, Yan Wu

**Affiliations:** 1 Department of Neurology, Union Hospital, Tongji Medical College, Huazhong University of Science and Technology, Wuhan, 430022 China; 2 School of Public Health, Tongji Medical College, Huazhong University of Science and Technology, Wuhan, 430030 China; 3 University of New Mexico Comprehensive Cancer Center, Albuquerque, 87131 USA; 4 School of Public Health, Shandong University, Jinan, 250100 China

## Abstract

Previous studies on the association between thyroid hormones and prognosis of acute ischemic stroke (AIS) reported conflicting results. We conducted a meta-analysis to assess the prognostic value of thyroid hormones in AIS. The PubMed, EMBASE, and Cochrane library databases were searched through May 12, 2017 to identify eligible studies on this subject. Out of 2,181 studies retrieved, 11 studies were finally included with a total number of 3,936 acute stroke patients for analysis. Odds ratio (OR) for predicting poor outcome or standardized mean difference (SMD) of thyroid hormone levels with 95% confidence intervals (95% CI) obtained from the studies were pooled using Review Manager 5.3. From the results, in AIS, patients with a poor outcome had lower levels of triiodothyronine (T3) and higher thyroxine (T4). Pooled OR confirmed the same association. Our study provides statistical evidence supporting the utility of thyroid hormone levels in prognosis of acute stroke.

## Introduction

Stroke, including ischemic stroke and hemorrhagic stroke, has become the second leading cause of mortality, and the third leading cause of disability, worldwide. In particular acute ischemic stroke (AIS), with a global prevalence of up to 299.1 per 100,000, causes a remarkable economic and social burden^1,2^. Meanwhile, thrombolytic therapy, as the only generally proven effective treatment for AIS, is seriously limited by its narrow therapeutic time window (<4.5 hours after onset) and high risk of the deadly adverse event, hemorrhagic transformation^3^. Survivors of AIS usually experience varied degrees of functional impairment or disability, meaning that long-term investment of manpower and financial resources is necessary to provide their health care and basic life support^4^. Therefore, it is of great importance to research the prognosis of ischemic stroke for use in the guidance of medical and rehabilitation therapy, and also to improve the life quality of stroke survivors. Several factors, including stroke severity, age, sex, vascular risk factors and comorbidities, have been found to be associated with the outcome of ischemic stroke. However, recent prognostic studies of other factors, like endocrine hormones and inflammatory cytokines, have not shown a consistent result^5–8^.

Thyroid hormones, mainly consisting of triiodothyronine (T3) and thyroxine (T4), have an irreplaceable role in the development, differentiation and maturation processes of brain tissue^9,10^. When triggered by thyroid stimulating hormone (TSH), the thyroid gland synthesizes and releases thyroid hormones, mainly T4. In the blood circulation, some of the T4 is converted into T3 by deiodinase. T3 shows three- to five-fold greater activity than T4. Both hormones appear in the peripheral blood in two forms: a free form and a bound form; the former enters the target cells and performs biological functions^8,11,12^. In recent years, decreased T3 concentrations have been found in AIS^12–22^ and in various other critical illnesses^23–27^, and an association has been shown with poor disease prognosis. In particular low T3 syndrome (low T3 level with normal thyroid TSH level), also called “euthyroid sick syndrome”, reflects dysfunction of the hypothalamus/pituitary/thyroid (HPT) axis and inhibition of conversion from T4 to T3 in the acute stress state. A number of clinical and fundamental studies have already focused on the association between thyroid hormones and AIS, but the conclusions appear inconsistent^8^. Thus, we were motivated to carry out this meta-analysis to estimate the prognostic value of thyroid hormones in AIS.

## Results

### Study characteristics

**Figure 1:**
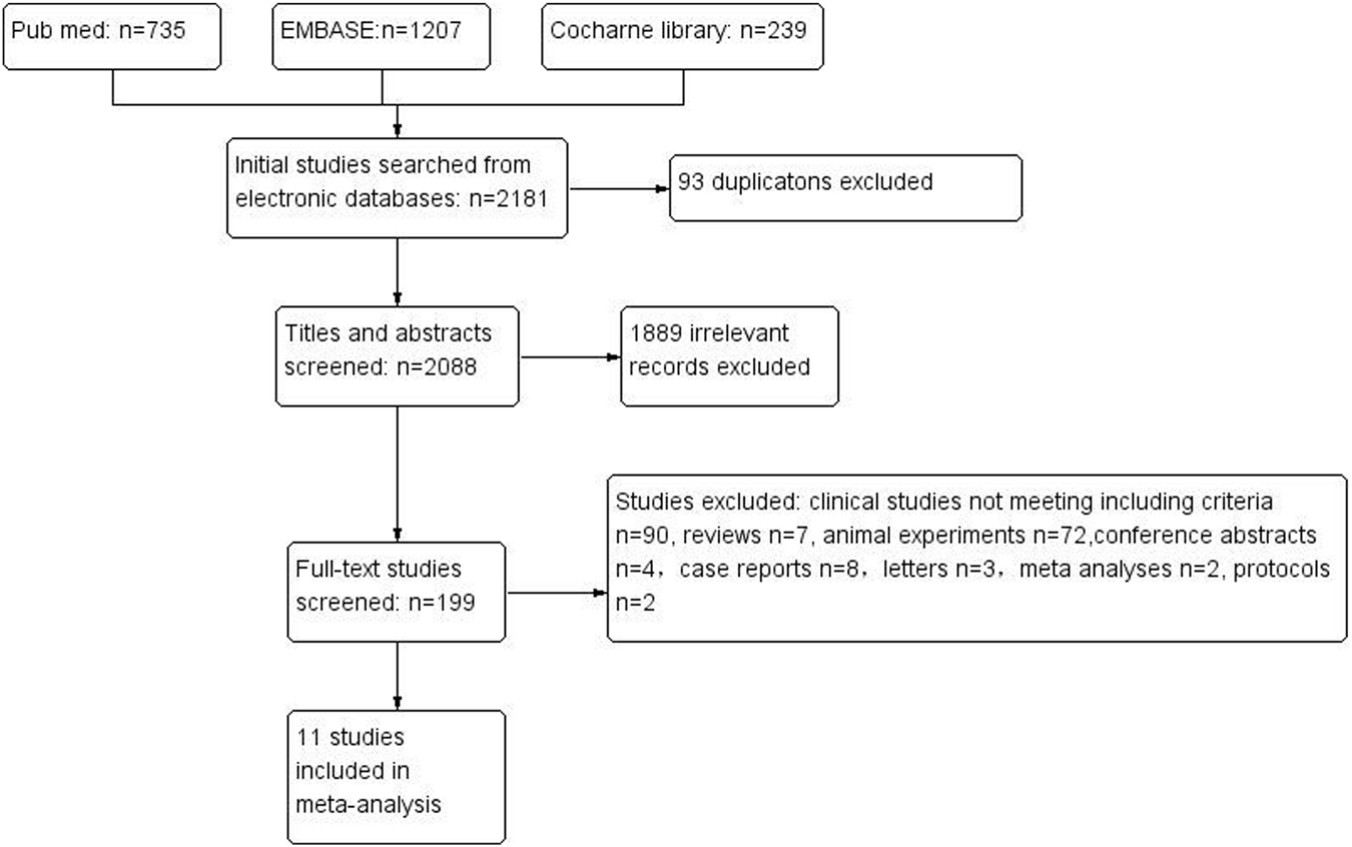
Flow diagram of studies selection.

Ultimately, 11 studies with a total of 3,936 patients were included in our meta-analysis^12–22^. The detailed process of initial study selection is shown in Fig. 1. The main characteristics of the included studies are presented in Table 1. Among them, five studies were conducted in Asia, four in Europe, and two in North America. One study focused especially on older people (mean age >80 years) and all participants aged >65 years^17^, while for any other study the mean/median age was <80 years. All studies evaluated prognosis using the modified Rankin scale (mRs)^28^, and 10 studies defined good outcome as an mRs score of 0–2 and poor outcome as a score of 3–6, while one study^22^ classified a good outcome as an mRs score of 0–1 and poor as a score of 2–6. In four studies^13,15,21,22^ that applied OR for prognostic evaluation, thyroid hormone level was presented as a categorical variable; while it was treated as a continuous variable in the other six studies. Sample sizes ranged from 47 to 775. The years of publication ranged from 2010 to 2016. The quality of the included studies was evaluated using the Newcastle–Ottawa Scale (NOS), based on which “high-quality” was defined as NOS ≥6.

**Table 1:** Main characteristics of studies in meta-analysis.

Study	Country	Sample size	Age and male,%	Follow-up time	NOS	Design
Wang Y 2016	China	359	63.12 ± 11.30years/73.6%	1 and 3 months	7	prospective
Suda S 2016	Japan	398	73.3 ± 11.9years/63.6%	at discharge	6	retrospective
Xu XY 2016	China	722	68(IQR59-76)years/61.1%	2–4 weeks after discharge	8	retrospective
Liu J 2016	China	46	63.6years/56.5%	at discharge	7	retrospective
O’Keefe LM 2015	America	129	67.03 ± 14.474years/60.5%	3 and 12 months	6	prospective
Forti P 2015	Italian	775	81.5 ± 7.6years/46.3%	at discharge	6	prospective
Cho HJ 2014	Korea	763	68(IQR 58–75)years/62.9%	3 months	6	prospective
Bunevicius A 2014	Lithuania	79	72 ± 11 years/65%	at discharge	5	prospective
Neidert S 2011	Switzerland	281	68(IQR 63–82)years/59%	3 months and 1 year	7	prospective
Ambrosius W 2011	Poland	337	68(years/59.2%)	1 month and 360 days	6	prospective
Zhang Y 2010	America	47	68.1 ± 12.7years/62.1% 66.8 ± 11.5years/61.1%	2–4 weeks after discharge	7	retrospective

### FT3 (serum free triiodothyronine)

**Figure 2:**
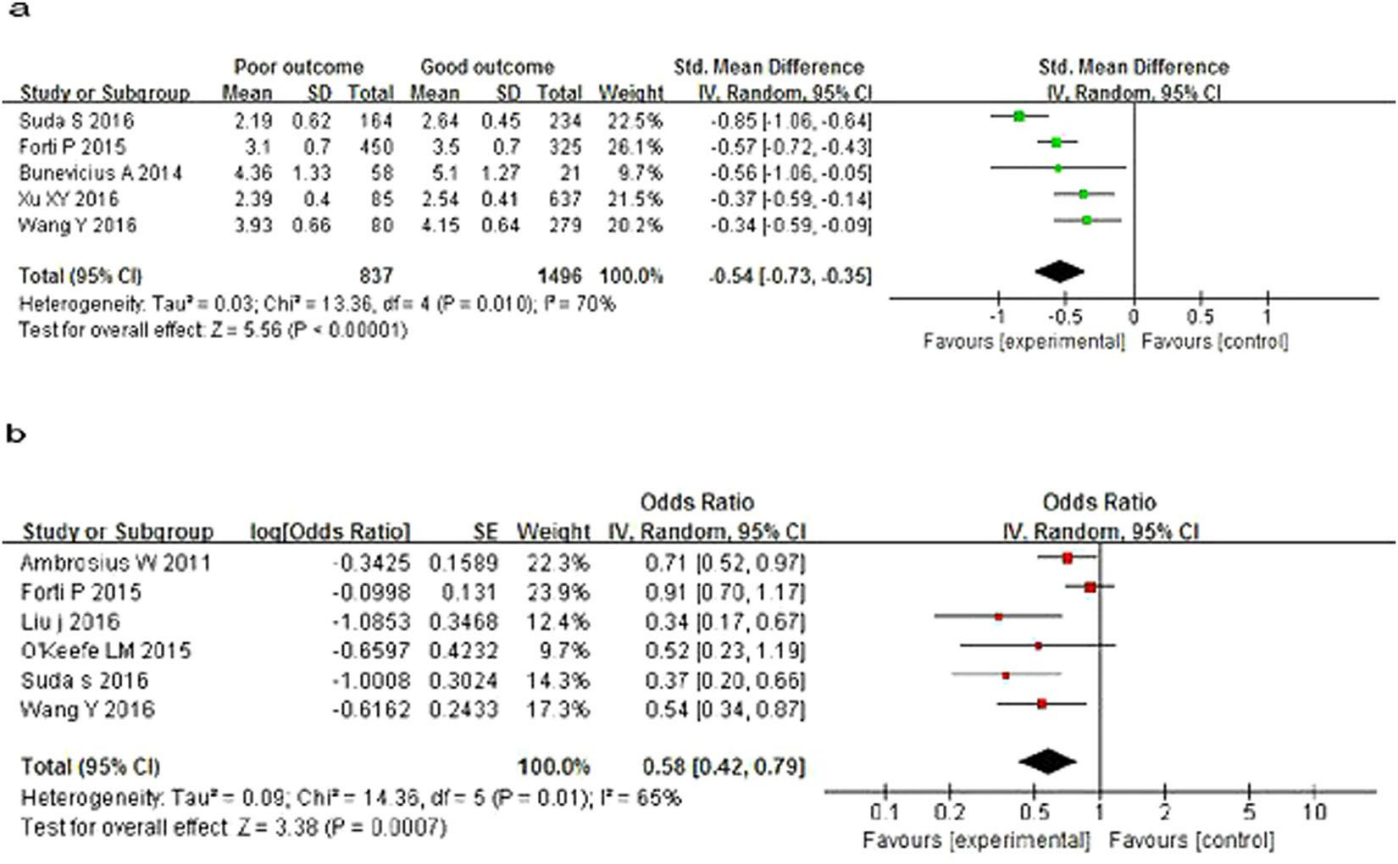
Forest plot of FT3 and prognosis of acute ischemic stroke.

Lower levels of FT3 were found in the poor outcome group [standardized mean difference (SMD) = −0.54, 95% CI = (−0.73, −0.35), *P* < 0.00001], with heterogeneity (*I*^2^ = 70%, *P* = 0.010; Fig. 2a). The pooled OR indicated a negative association between FT3 level and risk of poor prognosis in AIS [OR = 0.58, 95% CI = (0.42, 0.79), *P* = 0.0007], with heterogeneity (*I*^2^ = 65%, *P* = 0.01; Fig. 2b).

### FT4 (serum free thyroxine)

**Figure 3:**
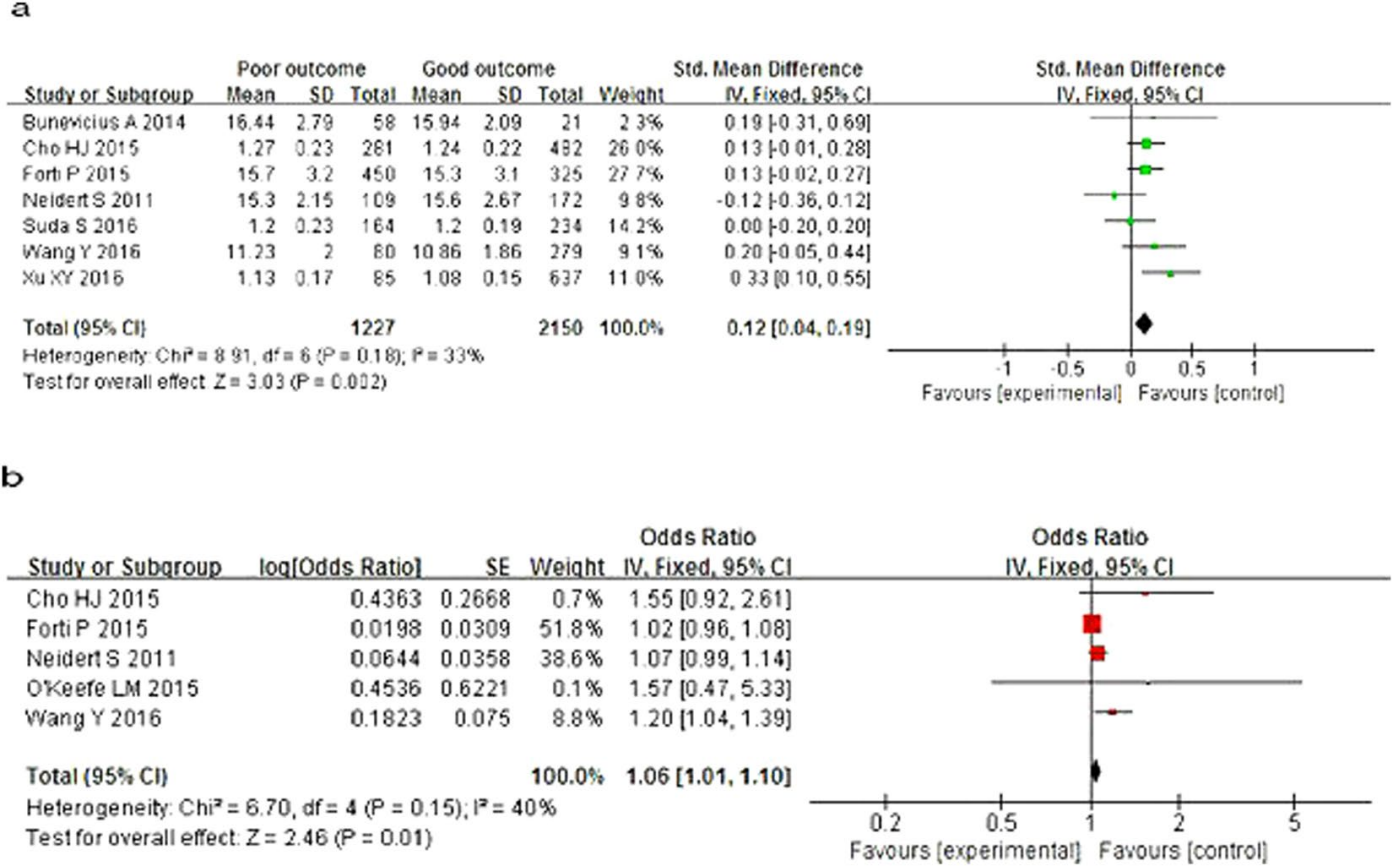
Forest plot of FT4 and prognosis of acute ischemic stroke.

Patients with poor outcomes had higher levels of FT4 [SMD = 0.12, 95% CI = (0.04, 0.19), *P* = 0.002], with heterogeneity (*I*^2^ = 33%, *P* = 0.18; Fig. 3a). The pooled OR also indicated a positive association between FT4 levels and risk of poor prognosis in AIS [OR = 1.06, 95% CI = (1.01, 1.10), *P* = 0.01], with heterogeneity (*I*^2^ = 40%, *P* = 0.15; Fig. 3b).

### TT3 (serum total triiodothyronine, including both free and bound forms)

**Figure 4:**
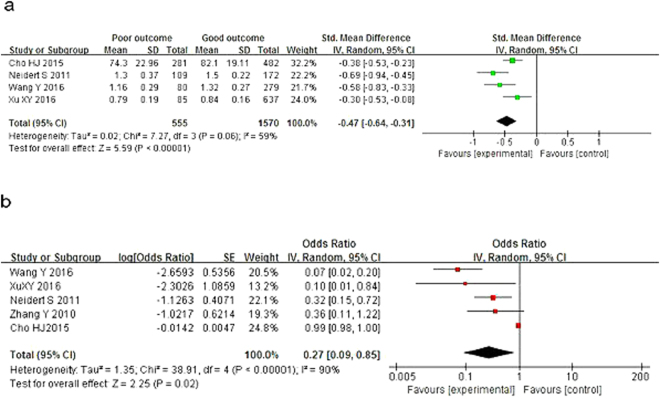
Forest plot of TT3 and prognosis of acute ischemic stroke.

The group with poor outcomes had lower TT3 levels [SMD = −0.47, 95% CI = (−0.64, −0.31), *P* < 0.00001], with heterogeneity (*I*^2^ = 59%, *P* = 0.06; Fig. 4a). The pooled OR implied a negative association between TT3 level and risk of poor prognosis in AIS [OR = 0.27, 95% CI = (0.09, 0.85), *P* = 0.02], with evident heterogeneity (*I*^2^ = 90%, P < 0.00001; Fig. 4b).

### FT3/FT4 ratio

**Figure 5:**
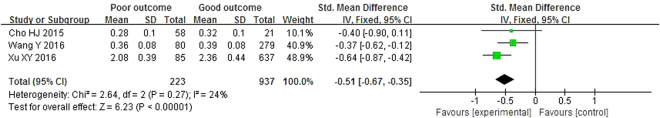
Forest plot of FT3/FT4 ration and prognosis of acute ischemic stroke.

Patients with poor outcomes had lower FT3/FT4 ratios [SMD = −0.51, 95% CI = (−0.67, −0.35), *P* < 0.00001], with heterogeneity (*I*^2^ = 24%, *P* = 0.27; Fig. 5).

### Subgroup analysis

Subgroup analysis was performed, taking into consideration the heterogeneous background of the patients in the studies. However, due to the limited number of studies, we only conducted a meta-analysis on the pooled OR of FT3 for disease prognosis. As shown in Table 2, with stratification by ethnicity, age and history of thyroid disease^29,30^, the conclusions remain significant among all groups and heterogeneity was reduced.

**Table 2:** subgroup analysis of FT3 and prognosis of acute ischemic stroke.

Analysis	Variable	NO.of studies	OR (95% CI)	Heterogeneity	Test for overall effect
FT3	all	6	0.58(0.42, 0.79)	*I*^2^ = 65%, P = 0.01	P = 0.0007
	Ethnicity				
	Asian	3	0.43(0.31, 0.60)	*I*^2^ = 0%, P = 0.44	P < 0.00001
	Caucasian	3	0.80(0.66, 0.97)	*I*^2^ = 20%, P = 0.28	P = 0.02
	Age(mean or median)				
	<80 years	5	0.56(0.45, 0.69)	*I*^2^ = 37%, P = 0.17	P < 0.00001
	>80 years	1	—	—	—
	History of thyroid disease				
	without	3	0.77(0.64, 0.93)	*I*^2^ = 49%, P = 0.14	P = 0.0
	uncertain	3	0.39(0.26, 0.57)	*I*^2^ = 0%, P = 0.72	P < 0.00001

### Publication bias

For analyses with at least five studies, we evaluated whether there was any publication bias. The measures used include Begg’s funnel plot and Egger’s test, given in Supplementary Fig. S1. Publication bias was found in two analyses.

Summarized results of the above meta-analysis are shown in Table 3.

**Table 3:** Summary of all analyses.

Outcome	Analysis	Studies	Result	Heterogeneity	Egger’s test
SMD	FT3	5	−0.54(−0.73, −0.35),P < 0.00001	*I*^2^ = 70%, P = 0.010	P = 0.778
	FT4	7	0.12(0.04, 0.19), P = 0.002	*I*^2^ = 33%, P = 0.18	P = 0.966
	TT3	4	−0.47(−0.64, −0.31), P < 0.00001	*I*^2^ = 59%, P = 0.06	—
	FT3/FT4	3	−0.51(−0.67, −0.35), P < 0.00001	*I*^2^ = 24%, P = 0.27	—
OR	FT3	6	0.58 (0.42, 0.79), P = 0.0007	*I*^2^ = 65% P = 0.01	P = 0.016
	FT4	5	1.06(1.01, 1.10), P = 0.01	*I*^2^ = 40%, P = 0.15	P = 0.096
	TT3	5	0.27(0.09, 0.85), P = 0.02	*I*^2^ = 90%, P < 0.00001	P = 0.023

## Discussions

Our meta-analysis demonstrated the predictive value of lower T3 levels for poor prognosis in AIS. As shown in Table 3, patients with poor outcome have significantly lower levels of FT3, TT3 and FT3/FT4 ratio, but higher FT4 levels. Results of pooled OR confirmed this association. Interestingly, T3 and T4 have a similar chemical structure and biological function, but they showed reverse associations with prognosis of stroke in our analyses. Nevertheless, this may not be a contradictory finding. In peripheral blood, most T3 (80–90%) is converted from T4 by deiodinase. This conversion could be inhibited in a clinical stress state. As a result, the level of T3 is likely to decrease while the level of T4 increases. T3 has three to five times greater activity than T4, so T3 is the major thyroid hormone playing widespread biological roles, including effects on the nervous system^8,11,12,14,20^. Consequently, in AIS, it may be the decline of T3 level that mainly affects stroke prognosis, even though the T4 level rises simultaneously.

As for the association between thyroid function and AIS mortality, four articles did study this separately^16,20,21,31^, while most others included “death” in the group of “poor outcome”. Among them, three studies^20,21,31^ showed a significant increase in mortality in patients with lower T3 or higher T4 levels. In contrast, one study^16^ found no association between T3 or T4 and AIS mortality. We did not conduct a meta-analysis on this subject due to the lack of sufficient studies. More clinical research is needed to confirm this association.

Several fundamental studies previously explored the possible mechanisms underlying the prognostic role of thyroid hormones in AIS. Some experiments found that exogenous T3 and T4 elevated the concentrations of certain important neuroprotective agents, such as brain-derived neurotrophic factor (BDNF) and glial cell-derived neurotrophic factor (GDNF), in a rat model of brain ischemia/reperfusion^32–35^. Two studies reported evident reductions of tissue infarction and edema in a transient middle cerebral artery occlusion (t-MCAO) model of male mice injected with T3, and attributed this anti-edema effect to suppression of the expression of aquaporin-4 (AQP4) water channels by T3^36,37^. Studies also found that T3 and T4 could inhibit apoptosis, resist excitability-related amino acid toxicity, suppress inflammatory reactions and increase ATP production by stimulation of astrocyte fatty acid oxidation in animal models of brain ischemia^34,35,38,39^. However, other experiments reported contradictory discoveries. One study observed lower release of glutamate, which causes toxic excitability of brain tissue, in ischemic gerbils rendered surgically or chemically hypothyroid^40^. Another study found a reduced neurological deficit, smaller infarct size, and fewer apoptotic neurons in hypothyroid ischemic rats^41^. Meanwhile two studies indicated that hyperthyroidism accelerated post-stroke injury in a rat model^42,43^. After reviewing the studies above, we must point out that in animal experiments^32–39^, the subjects were given exogenous T3 or T4 within hours of the induction of brain ischemia to explore the influence of thyroid hormone variation during the acute ischemic period, but in other studies^40–43^ hypothyroidism/hyperthyroidism was induced weeks before the establishment of the ischemic model, which thus involved the previous cumulative effect on the nervous system of abnormal thyroid function. Disappointingly, we only found one study that conducted a clinical trial on human patients, the results of which suggested that the use of thyroliberin (TRH) can inhibit “low T3 syndrome” and alleviate neurological deficit in the acute stage of ischemic stroke^44^.

One thing which should be noted is that hyperthyroidism, diagnosed as a level of T3 above the reference range, has been reported to increase risk of ischemic stroke, poor outcome in stroke and mortality in population-based studies^45–48^. The studies mentioned also indicated that preexisting hyperthyroidism had a thyrotoxic effect on ischemic brain tissue^42,43^, while hypothyroidism had a neuroprotective role^40,41^. Among the 11 studies included in our meta-analysis, seven studies excluded patients who had a history of thyroid disease. Therefore, the conclusion of our analysis–that lower T3 level predicts poor prognosis in AIS–is likely to be more applicable to patients who have no history of previous thyroid disease.

A just-published meta-analysis by Dhital *et al*.^49^, with a different analytical design and including different studies, also suggested a trend towards poor prognosis in stroke patients with lower T3 levels. However most of their pooled outcomes were not significant, probably due to the lower statistical power.

There are several limitations to our meta-analysis. (1) The number of studies finally included is limited, which would lower the power and precision of our results. (2) Heterogeneity in some analyses was high, and we were unable to accurately explore the source of heterogeneity for the limited number of studies. (3) We only included studies with prognosis classified as good or poor by mRs to improve the comparability of results. This single judgment for prognosis of ischemic stroke cannot be comprehensive enough, but it is the most widely-used around the world^28,50,51^. (4) Some non-significant data were not presented in our meta-analysis as they were not given in the original articles or not published at all, which would cause publication bias in some analyses.

In summary, our meta-analysis revealed an association between lower T3 levels and poor prognosis after AIS. More studies, particularly randomized controlled trails, are needed to verify whether thyroid hormone intervention can improve the prognosis after AIS.

## Methods

### Literature search strategy

The databases of PubMed, EMBASE, and the Cochrane library were searched to identify studies on the association between thyroid hormones and the prognosis of AIS up to May 12, 2017, using the following key words “stroke OR cerebral ischemia OR cerebral infarction OR cerebrovascular” AND “T4 OR thyroxine OR thyroxin OR tetraiodothyronine OR T3 OR triiodothyronine OR trilute”. Studies published in English were eligible for inclusion. The reference list of every identified publication was also checked manually.

### Study selection

All studies included had to meet the following criteria: (1) designed as a cohort study, nested case–control study or cross-sectional study; (2) the concentrations of thyroid hormones were measured within 72 hours after the onset of ischemic stroke^20,52^; (3) having definite inclusion criteria, including CT or MRI examination for acute ischemic stroke; (4) outcomes of ischemic stroke were assessed at discharge or follow-up within 3 months and were reported and classified as good/poor according to the Modified Rankin Scale (mRs); (5) odds ratio (OR), hazard ratio (HR), or relative risk (RR) for the outcome of ischemic stroke were reported or calculated from original articles. Mean and standard deviation (SD) or median and interquartile range (IQR) of thyroid hormones were given in both good and poor outcome groups. If a study presented outcomes at different follow-up times, we chose the data from the shorter term, because previous studies have reported that concentrations of thyroid hormone changed during the early period of AIS^20,52^, and longer follow-up would involve more confounding factors. If a study assessed adjusted and unadjusted OR simultaneously, we chose the latter since different adjusted factors would reduce the comparability of studies included. The exclusion criteria were: (1) studies with insufficient data for extraction; (2) animal studies, reviews, letters, case-reports, meeting abstracts; (3) articles published in languages other than English.

### Data extraction and quality assessment

Two of the authors (Chen JX and Wang J), extracted the following information from all original studies: first author, publication date, study type, country, characteristics of cohort, measuring time and method used for thyroid hormones, follow-up time, outcomes and adjusted factors. The quality of each article was evaluated using the Newcastle-Ottawa Scale (NOS)^53^, according to which “high-quality” is defined as NOS ≥ 6. Any differences of opinion were finally settled by discussion to ensure data accuracy.

### Statistical analysis

In this meta-analysis, all studies used ORs for risk estimates. We uniformly transformed them into ORs which evaluate the effect of increased hormonal level on poor outcome. An OR >1 indicates an increased risk of poor prognosis^54^. SMD was used to assess differences in the thyroid hormone levels between groups with poor and good outcomes. If studies reported the data as median and IQR, we estimated mean and SD using the median and the estimator SD = IQR/1.35^55^. Between-study heterogeneity was tested by *I*^2^ tests and Cochran’s Q statistic. *I*^2^ > 50% or *P* < 0.10 indicate high heterogeneity. The fixed-effect model was applied when *I*^2^ < 50% and *P* > 0.10. In contrast, random-effect model was used when *I*^2^ > 50% or *P* < 0.10^56,57^. Subgroup analysis was conducted to identify possible sources of heterogeneity. A two-sided *P* value < 0.05 indicates statistical significance. We conducted the above analyses using Review Manager 5.3 software. Publication bias was evaluated using Begg’s funnel plot and Egger’s test with STATA 11.0 software.

### Data availability statement

The datasets generated or analysed during the current study are available from the corresponding author on reasonable request.

## Electronic supplementary material

Supplementary Information - Begg’s funnel plot
