# Author Correction: Prognostic value of thyroid hormones in acute ischemic stroke – a meta analysis

**DOI:** 10.1038/s41598-018-24995-8

**Published:** 2018-04-24

**Authors:** Xingjun Jiang, Hongyi Xing, Jing Wu, Ruofei Du, Houfu Liu, Jixiang Chen, Ji Wang, Chen Wang, Yan Wu

**Affiliations:** 1 Department of Neurology, Union Hospital, Tongji Medical College, Huazhong University of Science and Technology, Wuhan, 430022 China; 2 School of Public Health, Tongji Medical College, Huazhong University of Science and Technology, Wuhan, 430030 China; 3 University of New Mexico Comprehensive Cancer Center, Albuquerque, 87131 USA; 4 School of Public Health, Shandong University, Jinan, 250100 China

Correction to: *Scientific Reports* 10.1038/s41598-017-16564-2, published online 24 November 2017

In the original version of this Article, there were errors in Affiliation 1 which was incorrectly listed as ‘Wuhan Union Hospital, affiliated to Tongji Medical College, Huazhong University of Science and Technology, Department of Neurology, Wuhan, 430022, China.’ The correct affiliation is listed below:

Department of Neurology, Union Hospital, Tongji Medical College, Huazhong University of Science and Technology, Wuhan 430022, China.

There were also errors in Affiliation 2 which was incorrectly listed as ‘Tongji Medical College, Huazhong University of Science and Technology, School of Public Health, Wuhan, 430030, China.’ The correct affiliation is listed below:

School of Public Health, Tongji Medical College, Huazhong University of Science and Technology, Wuhan 430030, China.

There were also errors in Affiliation 3 which was incorrectly listed as ‘University of New Mexico, Comprehensive Cancer Center, Albuquerque, 87131, USA.’ The correct affiliation is listed below:

University of New Mexico Comprehensive Cancer Center, Albuquerque 87131, USA.

There were also errors in Affiliation 4 which was incorrectly listed as ‘Shandong University, School of Public Health, Jinan, 250100, China.’ The correct affiliation is listed below:

School of Public Health, Shandong University, Jinan 250100, China.

These errors have now been corrected in the PDF and HTML versions of the Article.

