# Supplementary material for: Prognostic value of thyroid hormones in acute ischemic stroke – a meta analysis
Source: Sci Rep. 2017 Nov 24;7:16256. doi: 10.1038/s41598-017-16564-2 (PMC5701186; doi:10.1038/s41598-017-16564-2)
Supplement: Supplementary file 1 — Supplementary Information - Begg’s funnel plot [file 41598_2017_16564_MOESM1_ESM.pdf]

Prognostic value of thyroid hormones in acute ischemic stroke --- a meta analysis

Authors: Xingjun Jiang[1+], Hongyi Xing[1+], Jing Wu[2], Ruofei Du[3], Houfu Liu[4], Jixiang Chen[1], Ji Wang[1], Chen Wang[1], Yan Wu[1*]

1 Wuhan Union Hospital, affiliated to Tongji Medical College, Huazhong University of Science and Technology, Department of Neurology, Wuhan, 430022, China

2 Tongji Medical College, Huazhong University of Science and Technology, School of Public Health, Wuhan, 430030, China

3 University of New Mexico, Comprehensive Cancer Center, Albuquerque, 87131, American

4 Shandong University, School of Public Health, Jinan, 250100, China

*Corresponding Author, E-mail: wuyan_whunion@163.com

[+]These authors contributed equally to this work

Supplementary Figure 1. Publish bias Begg's funnel plot (a: FT3 SMD; b: FT3 OR; c: FT4 SMD; d: TT3 SMD; e: TT3 OR)

a

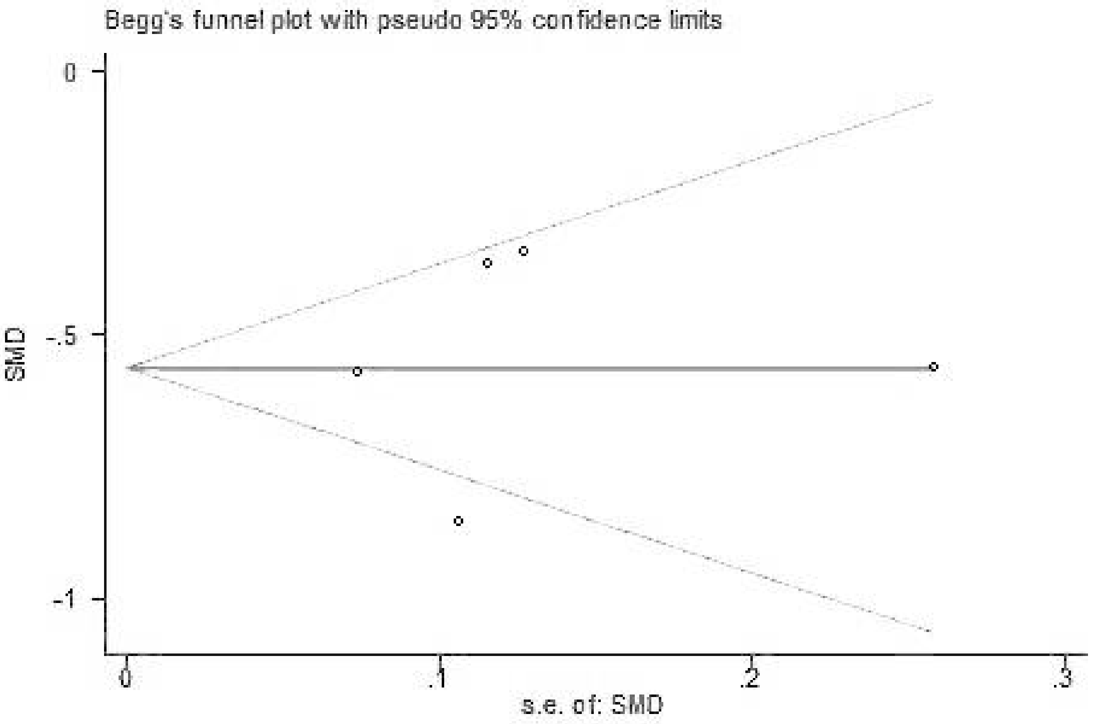

b

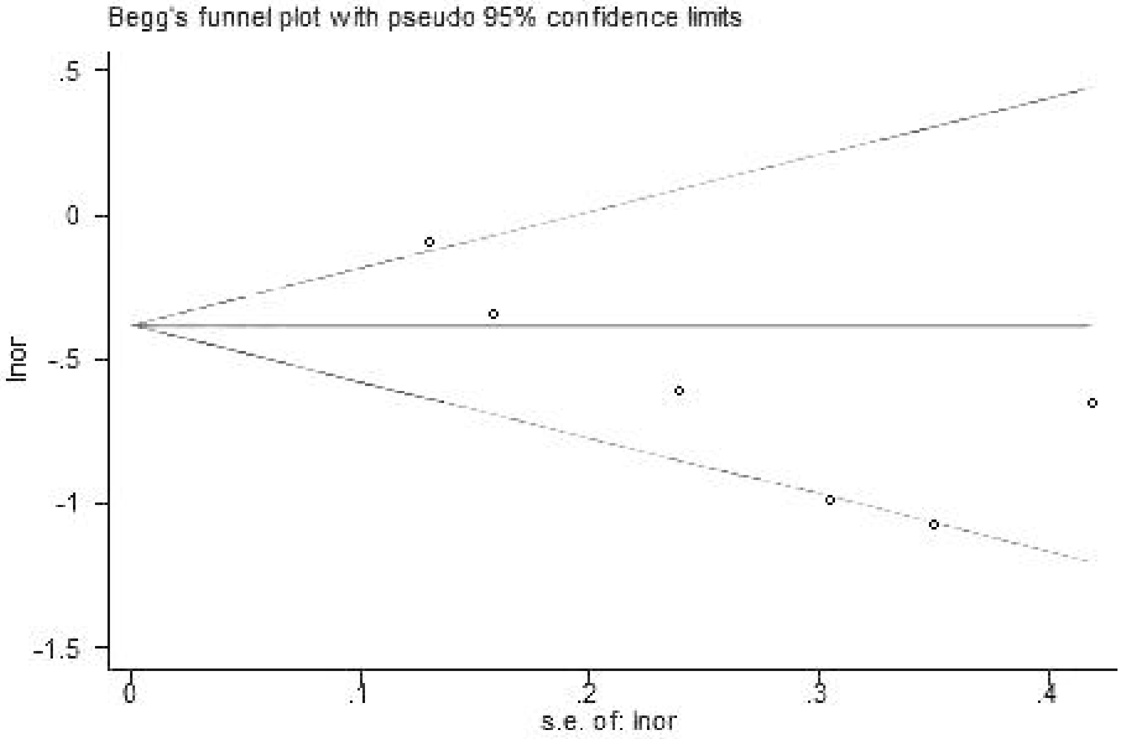

c

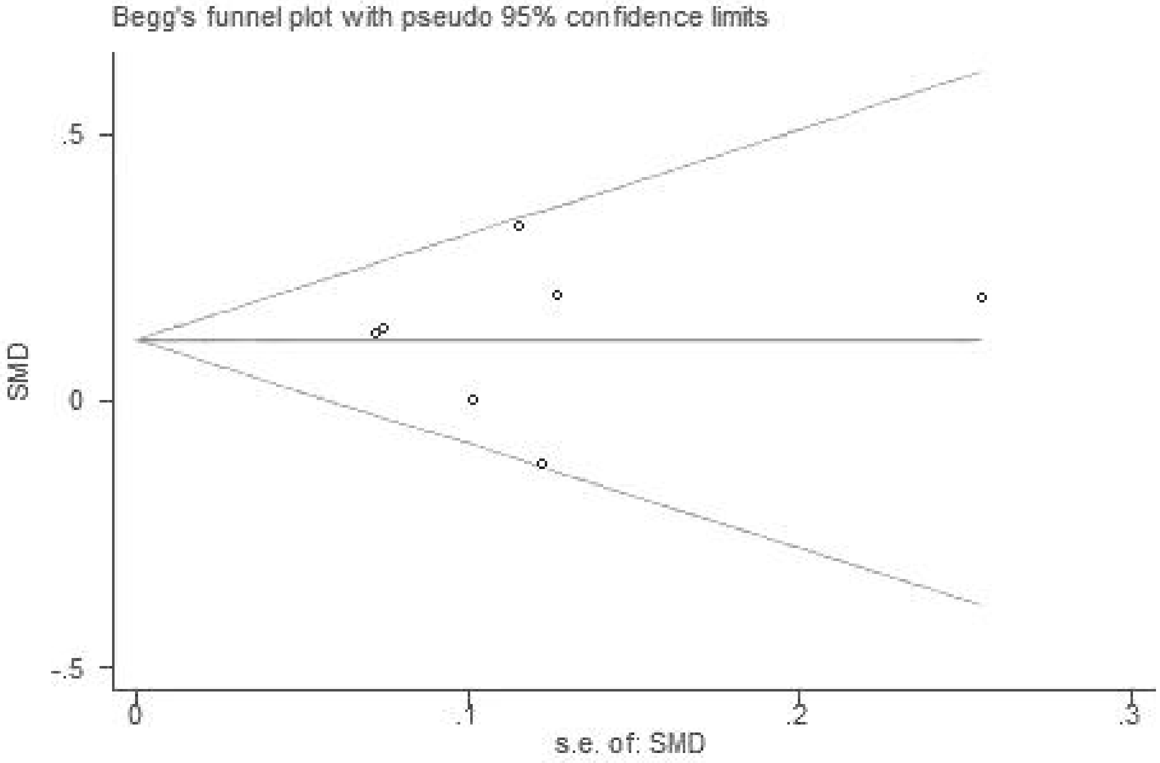
Begg's funnel plot with pseudo 95% confidence limits
SMD
.5
0
-.5
0
.1
.2
.3
s.e. of: SMD

d

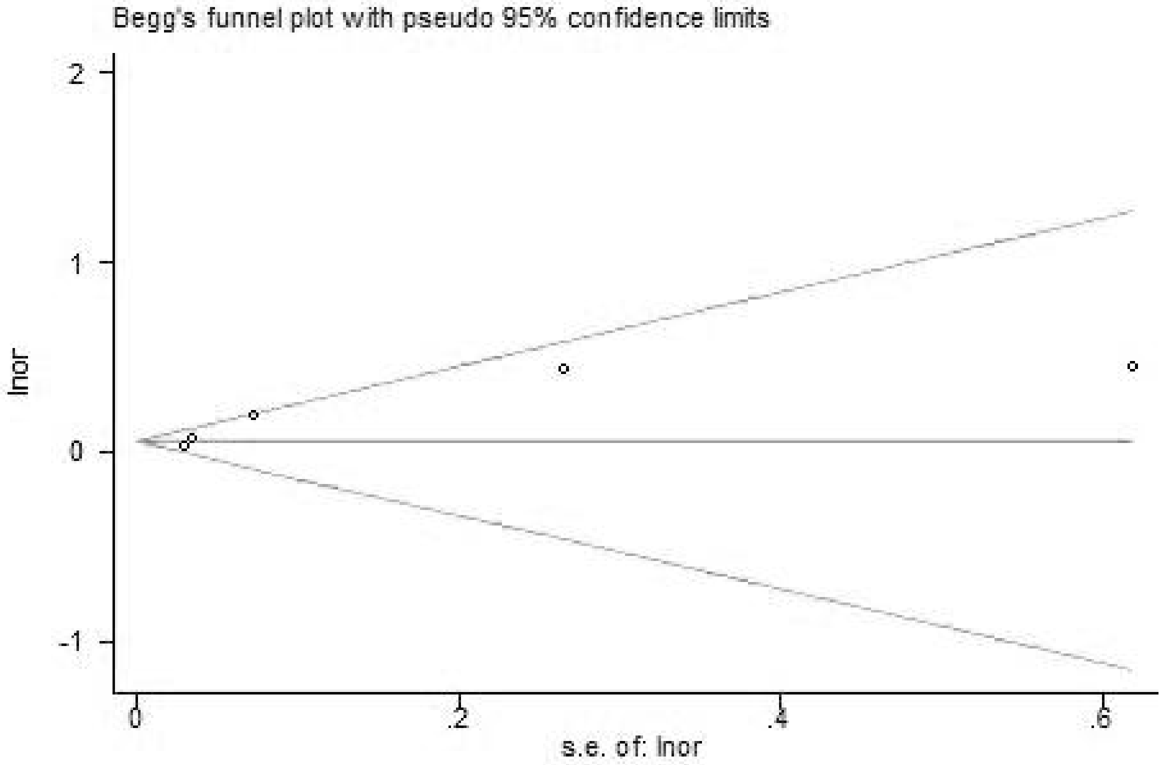
Begg's funnel plot with pseudo 95% confidence limits
lnor
2
1
0
-1
0
.2
.4
.6
s.e. of: lnor

e

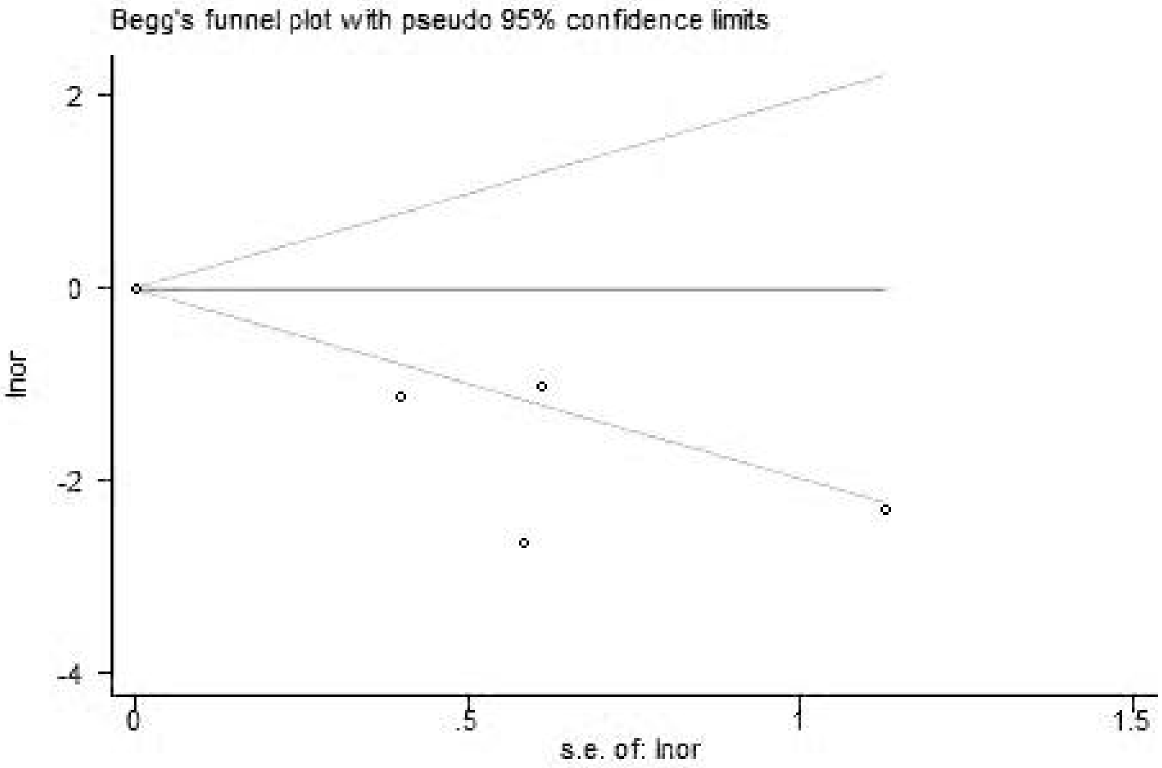

Begg's funnel plot with pseudo 95% confidence limits
2
0
-2
-4
lnor
0
.5
1
1.5
s.e. of: lnor
